# Changes in social mixing and attitudes and practices to precautionary measures in a maturing COVID-19 pandemic in six communities in Sudan: a qualitative study

**DOI:** 10.1186/s12889-024-18274-7

**Published:** 2024-03-26

**Authors:** Salma A.E. Ahmed, Rahaf AbuKoura, Abd Elhameed Ahmed, Omama Abdalla, Omnia Kamal Hassan, Ahmed Tom, Ahmed Eldirdiri, Drieg Ismaeil, Israa Zainalabdeen, Nazik Nurelhuda, Aljaile Ahmed, Afrah Abdan, Maysoon Dahab, Nada Abdelmagid

**Affiliations:** 1 Independent public health researcher, Khartoum, Sudan; 2 London School of Hygiene and Tropical Medicine, Department of Infectious Disease Epidemiology, London, UK; 3 Y-PEER Sudan, Khartoum, Sudan; 4 University of Khartoum, Khartoum, Sudan; 5 The Federal Ministry of Health, Khartoum, Sudan; 6 Sudan COVID-19 Research Group, Khartoum, Sudan

**Keywords:** COVID-19, Social distancing, Social mixing, Non-pharmaceutical interventions, Resource-poor, Participatory analysis, Sudan

## Abstract

**Introduction:**

With low COVID-19 vaccination coverage, non-pharmaceutical interventions were critical to mitigating the COVID-19 pandemic in Sudan. We explored changes in social contact patterns, risk perception, attitudes, and practices toward protective measures during an evolving COVID-19 outbreak in six illustrative communities in Sudan.

**Methods:**

This qualitative study took place in six communities in five Sudanese states using focus group discussions with community members and non-participant structured observations in public spaces between March 2021 and April 2021. A total of 117 participants joined 24 group discussions. We used a two-stage thematic analysis.

**Results:**

The perceived importance of compliance with individual preventative measures among those who believe in COVID-19 was higher than observed compliance with behaviors in most study sites. Adherence was consistently low and mainly driven by enforced movement restrictions. As restrictions were lifted, social contacts outside the household resumed pre-COVID-19 levels, and risk perception and individual and institutional adherence to protective measures diminished. We identified an environment that is socially and economically unsupportive of preventive practices, compounded by widespread rumours, misinformation, and mistrust in the government-led response. However, we identified new social habits that can contribute to reducing COVID-19 transmission.

**Conclusion:**

The unfavourable social and economic environment, coupled with the low visibility of the pandemic and pandemic response, has likely modulated the influence of higher risk perception on adopting precautionary behaviours by individuals. Governments and non-governmental actors should increase the visibility of the pandemic and pandemic response, enforce and incentivise infection control measures in public areas, promote emerging preventive social habits, and actively track and address rumours and misinformation related to COVID-19 and COVID-19 vaccines.

**Supplementary Information:**

The online version contains supplementary material available at 10.1186/s12889-024-18274-7.

## Background

The Sudanese national response to COVID-19 has largely fallen short in addressing the challenges posed by successive waves, as official figures for both incidence and mortality are widely recognised as significantly underestimated [1]. Literature reports suggest that only 0.2% of coronavirus infections were identified in Sudan, echoing the significant underestimation of both the prevalence and incidence of COVID-19 across Sub-Saharan Africa [2]. Soon after the first COVID-19 case in Sudan was confirmed on 13 March 2020, the government quickly declared a series of restrictions including school and university closures, bans of mass gatherings, international border closures and partial curfews, culminating with a complete lockdown between April and July 2020 [3, 4]. According to Sudan’s Ministry of Health COVID-19 situation reports, the second and third waves peaked in December 2020 and April 2021 [5], respectively. However, the national response beyond the first wave primarily relied on promoting individual protective measures, and no wide-scale movement restrictions were enforced. This stems from the fact that the initial imposition of movement restrictions has been widely criticised, primarily due to their adverse effects on the economy and the disruption of essential health services, despite their role in decreasing the incidence of COVID-19 [6–10]. The lockdown has also greatly hampered access to public health services, routine vaccination programs and the livelihoods of those at the margins of society [11].

Against this background, non-pharmaceutical interventions and preventative measures were the cornerstone of efforts to mitigate COVID-19 in Sudan and similar resource-limited settings [12–14]. This is especially important considering limited COVID-19 vaccination access and uptake [15, 16]. Among non-pharmaceutical interventions, social distancing and mask-wearing have been specially promoted as an alternative to wide-scale movement restrictions [12]. However, their acceptability and uptake in resource-limited settings are subject to desensitisation to infection risk [17]. For example, self-reported adherence to individual COVID-19 preventive measures overall among 1,371 individuals in Sudan September 2021 was 42%, with only 49% reporting avoiding physical greetings and handshakes [11]. Self-reported compliance with avoiding places of worship, public gatherings and staying at home was 41%, 58% and 38% respectively, with a consistent downward trend in successive surveys conducted since August 2020 [18]. Key barriers to the uptake of key protective behaviours in Sudan are poorly understood. However, financial and logistical barriers, high levels of stigma, denialism, and misinformation are all suspected to play a role [19, 20]. The effectiveness of future COVID-19 mitigation efforts relies on understanding and addressing these barriers to increase the acceptance and feasibility of key preventative measures [21].

To our knowledge, there are no published studies that explored social contact patterns in Sudan in general and in the targeted communities in particular. Understanding how different population groups mix and interact socially during the pandemic is a crucial indicator of how viral transmission will occur and critical in informing social distancing interventions tailored to contact patterns unique to Sudanese contexts. It will also allow a more rigorous assessment of the effectiveness of different social distancing interventions. This study explored changes in social contact patterns relevant to COVID-19 transmission and the extent, barriers, and enablers of adherence to recommended protective behaviours and measures during the COVID-19 outbreak in six illustrative communities in Sudan. We aimed to inform policymakers and non-governmental COVID-19 responders of appropriate adaptations and contextualisation of social distancing interventions to encourage higher uptake in the targeted communities.

## Methods

### Study design

This is an exploratory qualitative study using a combination of focus group discussions (FGDs) and non-participant structured observations in public spaces. The design draws on the protection motivation theory [22], which assumes that an individual’s decision to adopt and comply with preventative health behaviours is based on their motivation to protect themselves from health threats after weighing the risks and potential benefits [23]. The FGDs provide insight into how people appraise the threat of COVID-19 to their health and their beliefs about the necessity and feasibility of the recommended behaviours (coping appraisal). On the other hand, non-participant observations were used to objectively identify adherence to protective behaviors, practices, and measures in public areas, and allow us to compare reported and observed adherence to protective measures in the study population.

### Study setting

We conducted the study in six illustrative communities in five Sudanese states, representing different settings. In Khartoum state, we chose Ombadda locality, a high-density poor urban population with pockets of long-term internally displaced populations, and Tuti Island, a close-knit agricultural community. We selected two other urban, and peri-urban cities in North Kordofan and Blue Nile states, El-Obeid and Damazin, respectively. In addition, we included one internally displaced persons’ (IDP) community in South Darfur, Dereij IDP camp, and one rural community in the south of Gezira state (Abu Haraz). Ombadda is a high-density poor urban community in Khartoum State, where the main sources of income are low-wage jobs and micro-businesses. The education level is generally high, particularly for girls, as boys tend to leave secondary school to support their families. Tuti Island is an island in the heart of the capital city Khartoum. Its inhabitants are close-knit and historically descend from a few families and tribes, and homes tend to house extended families. The community is a low- to -middle economic one, where most household incomes coming from freelance trade and businesses. Inhabitants typically have high educational levels. El-Obeid is an urban middle-class community and the capital of North Kordofan state. Most residents work as public servants or traders. Our study site in Damazin, the capital city of Blue Nile state, is a poor peri-urban community, where most residents work as manual laborers, small-scale farmers or traders. Abu Haraz village is a rural village on the east bank of the Blue Nile river and is located approximately 17 km from Wad Madani, Gezira State’s capital. Its inhabitants are mainly farmers, and Abu Haraz is host to a large Sufi community and Sufist shrines. Dereij IDP camp is situated 4 km from Nyala city, the capital of South Darfur state. Its residents are long-standing IDPs, mostly poor and dependent on humanitarian aid.

Sudan’s Youth Peer Education Network (Y-PEER Sudan) was actively engaged in promoting COVID-19 prevention, through its member volunteers, or “Y-peers”, in the sites at the time of the study. The selection of communities was mainly operational based on the accessibility of the community (existence of Y-peers in a specific community) and the existence of activities of awareness-raising campaigns as the research was meant to inform these interventions.

The Sudan COVID-19 Research Group, in partnership with trained volunteers from Y-PEER Sudan, a nationwide network of youth volunteers and researchers, designed and implemented the study. Since the start of the pandemic in Sudan, Y-PEER Sudan has implemented COVID-19 research, advocacy and awareness-raising activities across Sudan. Y-peers were community members living and working in the study sites.

We trained male and female Y-peers remotely to collect qualitative data through live and pre-recorded short audio/video training sessions via WhatsApp and YouTube. The Y-peers received training on study procedures (data collection tools and informed consent process), qualitative research, and preventative strategies developed and delivered by co-authors NA, SAEA, and RA. Each training session was followed by a live questions & answers online session with trainers, and a WhatsApp group was used to facilitate communication and provide ongoing support. We asked Y-peers to pilot the FGD guide and observation checklists in their communities as part of the interactive training. A weekly online meeting took place to troubleshoot and share updates on data collection and field experiences.

### Study participants

Y-peers purposively identified eligible participants for the FGDs through their existing community contacts. Participants were approached by the Y-peers either face-to-face or through the phone. At each study site, four FGDs were conducted: adolescent/young women, adolescent/young men, adult women, and adult men, to achieve a balanced sample of age and gender among participants. Inclusion criteria for FGD participants included being males or females aged 18–24 years (younger women and men) or 25 − 59 years (older women and men) and living in the study community. We excluded the following: those who did not provide informed consent, those aged less than18 years or more than 59 years of age, those who lived in the same household as another participant selected for the study, persons of any age with bronchial asthma, diabetes, hypertension or have immunosuppression, and those who had COVID-19-like symptoms in the past two weeks. Those older than 59 were excluded as they are at higher risk of severe COVID-19, and we did not want to expose them to the risk of COVID-19 transmission during FGDs. Each FGD session comprised 4 to 8 participants. Youth peers purposively identified initial eligible participants through their existing community contacts and used snowball sampling to capitalise on naturally occurring social networks in the community to construct the focus groups. This ensured we minimize exposure to COVID-19 through bringing people together into a focus group who were already in regular social contact with each other. It also ensured that groups were fairly homogenous in socioeconomic status, as neighbours, friends and family members. All those who were invited to participate joined the FGD sessions and no refusals or drop-outs occurred.

The places for structured observations of public spaces were purposively selected to reflect various functions, layouts, outdoor versus indoor settings and use by different population subgroups. In each study site, observation locations were a public transportation journey, a place of worship, the nearest health facility or pharmacy, the local open market, and a bakery. We selected the place of worship, health facility or pharmacy, and bakery known to be most used by the study population or closest to the geographical centre of the study site, after discussion with existing community contacts. We also observed the closest local open market, which in Sudan is most commonly one market serving a large neighborhood, or one market serving several small neighborhoods. For public transportation observation, the Y-peers took a 15- to 30-minute journey on any local transportation vehicle departing from the study community between 10 am and 4 pm.

### Data collection

The data collectors conducted four FGD sessions with 6 to 8 participants per session at each study site. Each site coordinator telephoned the eligible participants identified by the volunteers. After receiving informed consent and permission for the group discussion to be recorded, the focus group discussions took place at a pre-agreed date, time, and location chosen by and convenient to participants. The FGDs happened within few days of recruitment of the participants (2 to 4 days). Data collectors used a pretested FGD guide to facilitate the discussion (Supplementary file 1). The FGD guide covered the following topics: [1] risk perception, [2] social contact patterns, [3] individual protective behaviors outside the home, [4] communal distancing measures, and [5] factors affecting compliance with preventative measures. Finally, participants were asked to recommend measures for encouraging and supporting people to successfully adopt and maintain protective behaviors outside the home for COVID-19 prevention. The FGD sessions were conducted in the Sudanese dialect of the Arabic language, the native language of the participants, interviewers and authors. The FGD sessions lasted between 40 and 80 min. The non-participant structured observations were conducted using set time and duration at pre-defined public locations using a checklist specific to each observation site (supplementary file 2). The observation checklist comprised of four sections: implementation of COVID-19 related health and safety measures, (ii) adherence to COVID-19 prevention behaviours, (iii) description of people present in the observed location, and (iv) a description of the environment at the observation site. The observation periods lasted between 20 and 30 min. We considered this sample size a manageable workload for the Y-PEER volunteers during an outbreak. Observations and FGD sessions were conducted in parallel between March 2021 and April 2021.

Before finalisation, the FGD guide and observation checklists were piloted in one focus group and two public spaces by two different data collectors to check for suitability of content, translation and sequence/flow of questions and duration of the data collection. After a preliminary analysis of data for each site, we reviewed the data and revisited the need to increase the number of group discussions or observations to reach data saturation.

### Data management and analysis

Audio-recorded interviews were transcribed verbatim in Arabic, and then transcripts were verified by the Y-peers for accuracy. Field notes and filled checklists from observation were typed in Arabic. All transcripts and checklist notes were analysed in Arabic. We conducted a preliminary analysis using a site-specific summary report prepared by each site team, summarising key themes, describing the context of data collection and identifying immediate insights. After that, the co-authors invited the observers and FGD facilitators to participate in a group discussion. We used the reports to prompt follow-up questions and use the interpersonal dynamic of group discussions to generate new insights. These FGDs with data collectors helped formulate initial and preliminary ideas and obtain the study team’s reflections on the extent to which supportive measures facilitated social distancing in public areas and to which people adhered to such measures. They also generated preliminary ideas for detailed analysis. The discussions were recorded and employed by the co-authors to develop preliminary findings report for quick dissemination to Y-PEER Sudan, the Ministry of Health, other COVID-19 responders and targeted communities. Y-peers organised sessions with the targeted communities to get their feedback on research findings as part of the awareness-raising campaigns.

We conducted an in-depth analysis of the transcripts and observation checklists using a thematic analysis approach based on the framework method [24]. We used deductive and inductive approaches to derive possible themes from study questions and discussion guides while allowing new themes to emerge from the data [25]. Data for each site was independently analyzed for possible themes by the co-authors SAEA, NA, RA, IZ, NN, and A Ahmed. NA then collated the analysis in a single spreadsheet and a narrative document. Co-authors reached a consensus on themes and subthemes, and categorisation was agreed upon. The themes were interpreted further and compared to the objectives of the study to generate conclusions. Explanatory and corroborative quotes were extracted by NA, translated into English by SAEA and back-translated to Arabic by RA. This article adheres to the Consolidated Criteria for Reporting Qualitative Research (COREQ) reporting guidelines [26] (Supplementary file 3).

## Results

**Fig. 1:**
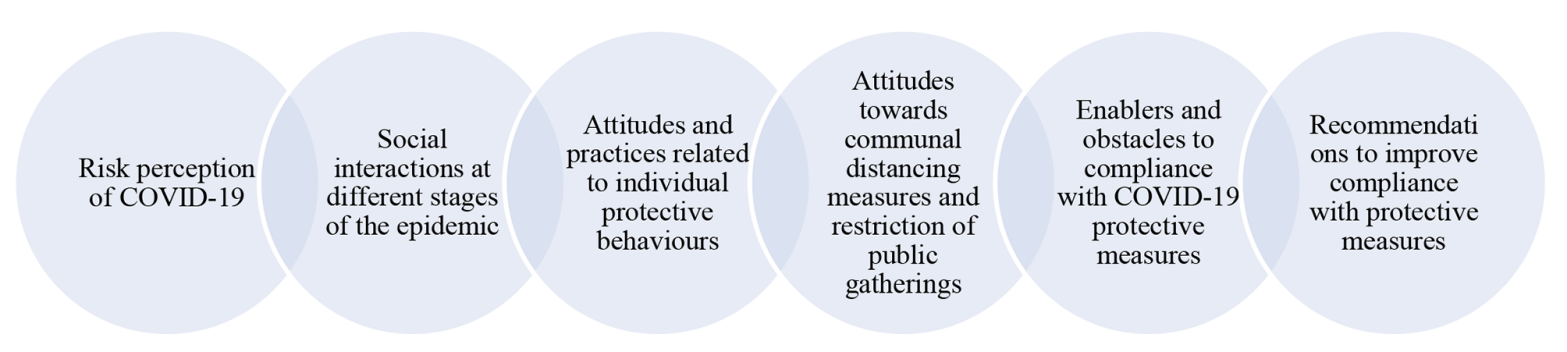
Main categories of study findings

A total of 122 participants attended 24 focus group discussion groups in the six study sites (El-Obeid, Tuti Island, Dereij IDP camp, Ombadda, Damazin, Gezira). The number of participants and age breakdown of FGDs is presented in Table 1. Our study aimed to explore the social interaction patterns during COVID-19, self-reported individual and communal protective behaviors adopted, and to understand the factors that affect compliance with recommended protective behaviours and measures during the COVID-19 outbreak in Sudan. We present the findings according to six broad categories (Fig. 1).

**Table 1:** FGD participants across the six study sites

Site	Number of participants				Age range of participants per group (years)			
	Younger women	Younger men	Older women	Older men	Younger women	Younger men	Older women	Older men
El-Obeid	4	4	5	4	22–24	19–24	28–54	25–30
Tuti Island	5	4	5	4	21–24	20–22	28–45	26–49
Dereij IDP camp	6	6	6	6	19–23	19–22	23–38	28–50
Ombadda	8	4	5	6	18–26	20–24	25–53	25–30
Damazin	5	6	6	4	19–29	18–24	28–39	25–59
Gezira	5	6	4	4	19–24	18–24	29–38	25–27

**Fig. 2:**
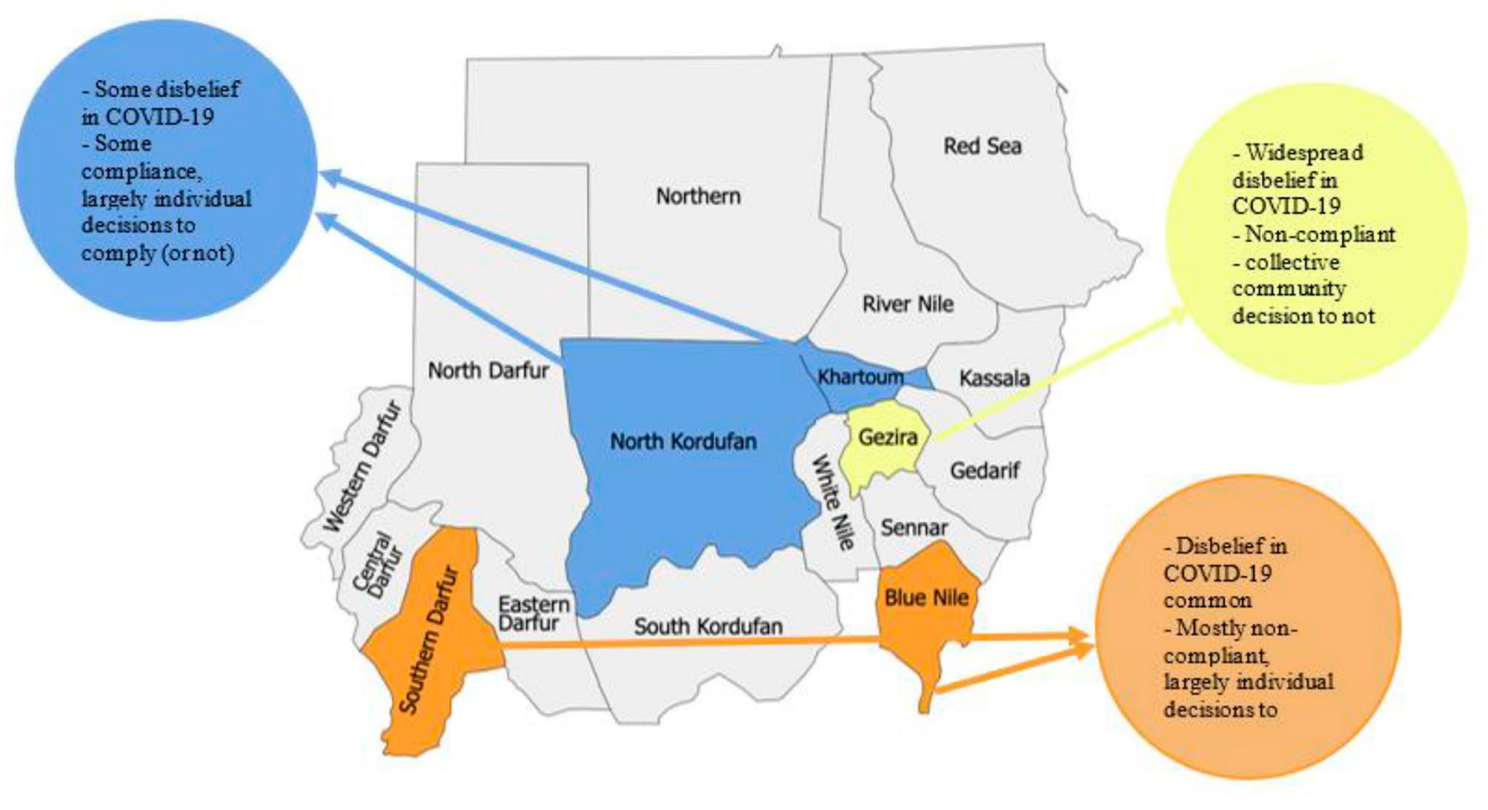
Grouping of study sites by similarity of findings

### Risk perception of COVID-19

Varying levels of risk perception were reported within sites, except for Gezira, where risk perception seemed consistently low throughout the pandemic, regardless of gender and age (Fig. 2).

Participants reported that risk perception decreased as the pandemic evolved and their perceived familiarity with the disease grew. However, in two sites (El-Obeid and Tuti Island), adult women expressed that worry increased once schools re-opened and government measures were lifted in July and August 2020 (Fig. 2).

*“In the beginning, I was worried, like 90% worried, as the media was a big driver of that fear; currently I am not worried because the situation is normal”* [younger man, Dereij IDP camp].

*“I was very worried about the illness, because I have seen people who were sick, and the number of cases was increasing. However, during the second wave, I was not worried at all”* [younger woman, Ombadda].

Concerns over the risk of COVID-19 seemed to vary by gender and age, with adult women seeming more concerned while adult men seemed least concerned.

*“W Allah [I swear] I worry a lot, especially for our offspring who work outside the house, we are staying at home. My son once had flu; I thought this would be corona [COVID-19]. I kept going to his room to check on him at night”* [older woman, Tuti Island].

Almost all participants reported worrying about others rather than themselves. Fear was driven by cultural tendencies to protect the family and the vulnerable, with participants mentioning concerns for the elderly, children, and those with chronic illnesses.

*“One needs to be careful because you have an old person at home. My father, may Allah grant him health and well-being, is sick. He has diabetes and hypertension. We interact with him in a way that protects him from infection, that means you shouldn’t be causing any harm to someone else”* [older man, Tuti Island].

Participants reported several factors that influenced the levels of risk perception at different stages of the pandemic. Increased COVID-19 cases and deaths reported by the Ministry of Health or observed in their local communities increased risk perception. In addition, the high-profile and prominent response at the beginning of the pandemic increased risk perception, for example, intensive media messaging and scaling up of isolation centres. Similarly, the fall in cases and deaths and the response’s waning have reduced risk perception.

*“I was afraid of severe illness Because I saw that the numbers were increasing, especially in Sudan. But during this second wave, I wasn’t afraid at all.”[Younger woman, Ombada].*

*“Various channels, including the media, the Ministry of Health, governmental initiatives, and the intensity of awareness-raising campaigns have dwindled. This is compounded by societal negligence. Consequently, there still exists people who deny the existence of COVID-19” [Older woman, Obaid]*.

Furthermore, as the disease became familiar over time, the severity of the disease, seemingly less than seen in other countries, has contributed to lower risk perception. Despite the prolonged nature of the pandemic, COVID-19 denialism was not uncommon.

*“Until now, I’m not convinced of the existence of COVID-19 in Sudan, and my lifestyle hasn’t changed, like socialising and handshaking”[younger woman, Damazin]*.

*“By God, I haven’t changed a thing. Believe me, I didn’t believe a word of it. I was doubtful whether there was even a coronavirus, to begin with. Every time I discussed it with people, I said, “Brothers, this whole thing they show on television is about sick people in America, and other Arab countries, etc. Hey, these things are not happening in Sudan, do you see anyone in Sudan?”* [older man, Tuti Island].

Participants reported that rumours about COVID-19 continue to drive disbelief in COVID-19 and low-risk perception.

*“ [they say] no, no, it’s just a normal flu,’ and they pass it on to the people with them.. This is nonsense; these are just government matters.”* [younger woman, Gezira].

*“Come on, the society itself feels there’s a lack of credibility from the government. Because in every COVID case, there was supposed to be [external] financial support [to the government], but where did the support go? I can’t believe any official claiming support didn’t come [from abroad].”* [younger woman, Damazin].

### Patterns of social interactions at different stages of the pandemic

For this study, relevant social contacts were either physical contact or a two-way conversation with three or more words within one meter of another person but no physical contact. Prior to COVID-19, prevailing cultural traditions included weddings and funerals that lasted for days or weeks, with people expected to attend daily and spend hours or days at these events. Generally, relevant social contacts outside the household decreased during the first wave when severe movement restrictions were enforced and gradually resumed pre-COVID-19 levels once the restrictions were lifted in July 2020. However, even after the first wave, women in Tuti Island and El-Obeid reported some sustained reductions in social contacts, such as staying less at social events such as weddings and funerals (Fig. 2). Also, and in line with consistently low-risk perception, hardly any changes in social contacts occurred at any pandemic stage in rural Gezira. During movement restrictions, this rural community collectively installed measures to maintain social interaction and bypass movement restrictions, e.g., opening a new ‘Corona’ market when usual markets were closed by public orders, continued mass prayers outside closed mosques and continued gatherings in homes rather than closed public social clubs (Fig. 2).

*“No change happened. Even in Eid, people went out and visited each other and everything.”*

[younger woman, Gezira]

Regardless of the stage of the pandemic, people tended to maintain the same pre-pandemic close social contacts with people they are familiar and comfortable with, such as family, friends and neighbors. Even during the lockdown, most close contacts in people’s immediate social circles remained unchanged.

*“The people whom I interacted with the most during COVID-19 time were those in my neighborhood. We were in lockdown, but we usually sat down in our street or played football close to sunset time. We sat outside until 11 pm or midnight and so forth”* [older man, Ombadda].

*“I mean, even in Ramadan, we used to pray in a group although it was not allowed to do so, we tended to pray with the people we could trust. You can see the person in front of you washing and sanitising his hands which means he may not get COVID-19, unlike those whom you meet in mosques– you don’t know their whereabouts”* [younger man, Tuti Island].

Participants reported changes in some different types of social contacts during periods of severe movement restrictions. Contacts in public facilities were reduced due to enforced closures and infection prevention measures in banks, pharmacies, and hospitals. Bans of large gatherings during the first wave also reduced contacts in social events, especially weddings and funerals. Although these events did not wholly stop despite the ban, they were adapted to be shorter and smaller. In fact, some respondents in Damazin reported that some people took advantage of the ban on gatherings to hold more celebratory events, thereby avoiding the higher costs of traditionally more extensive and longer events. Respondents also reported that contacts related to economic and educational activities were reduced during periods of severe restrictions when schools, markets and public services were either closed or operated at reduced hours. A few respondents also reported that informal social gatherings in the neighborhood, such as regular pre-pandemic coffee meetups and social house visits between families and neighbours, became smaller, shorter and less frequent. In addition, there was a reduction in the frequency and number of attendees of informal neighborhood social gatherings, which before the pandemic, would have been daily or weekly gatherings between neighbours, friends or extended family members.

Sudanese culture is strongly collectivistic, with a very strong sense of community, mutual reliance and a sense of duty towards relatives and neighbours. An individual’s social capital is, to a large extent, determined by the extent to which they participate in their community. In our study, participants reported that the primary influence for reducing social interactions outside households was government enforcement of movement restrictions rather than a personal drive to reduce contacts. However, a few participants found the enforced measures a welcomed opportunity to reduce social contacts without losing social capital. They felt conflicted between a sense of necessity to protect themselves from COVID-19 and a firm rejection of restrictions by people in their social circle.

*“I personally see it as a considerable change. We are not the kind of people who live according to a specific pattern of social interaction. For me, socialising is something that comes naturally or spontaneously. We did not have any previous experience with social reduction or isolation, and this was the first time for us to experience such measures. On a personal level, I exerted a lot of effort to get used to this idea. Although deep down I know the necessity of these precautions and the severity of the illness, I find myself conflicted between these personal beliefs of the need for isolation and the societal and family customs.”* [younger man, Damazin].

### Perceived importance and adoption of COVID-19 protective behaviors

Individual protective behaviors explored in this study include avoiding physical greetings, wearing face coverings, handwashing, sanitising, physical distancing, avoiding contact with people with COVID-19-like symptoms and vaccination. Our findings indicate the perceived importance of compliance among those who believe in COVID-19 is higher than reported and observed compliance with behaviors in most study sites, but especially in Tuti Island and Ombadda. Based on observation of public spaces in study areas, there were varying levels of compliance with individual protective behaviors within sites (except Gezira, which showed hardly any compliance), but a generally low level of compliance across sites (Fig. 2). A detailed description of the adoption of COVID-19 individual protective measures is illustrated in Table 2. Participants discussed that abstaining from physical greetings was, by far, the most challenging behavior to comply with, even among those worried about COVID-19. Politeness is strongly embedded in Sudanese culture, and therefore participants reported that abstaining from physical greetings presented the most significant social challenge, as well as being a habit that is difficult to break. As a result, physical greetings continued even if it meant incurring additional financial costs of using hand sanitizer after handshakes.

*“I cannot embarrass girls who want to greet or say to them physically do not shake hands with me because of corona. I just sanitize my hands after handshaking”* [younger woman, Ombadda].

*“As our brother mentioned, women did not abandon handshake and physical greetings– everywhere! In the market or street, 24/7, mostly with hugs and kisses and the like”* [older man, Ombadda].

At the time of data collection, COVID-19 vaccination rollout in Sudan had begun for high-risk groups in 2 of the 5 states in which there were study sites (Khartoum and Gezira). At the time, there were no publicly-declared plans to offer the vaccine to the wider population. Participants expressed various attitudes towards COVID-19 vaccination, including welcoming attidues, scepticism about benefit versus harm, and refusal of vaccination. Some respondents reported rumours circulating in their communities of the side-effects of the vaccine.

*“Personally, if the [COVID-19] vaccine is [offered to me], I will get vaccinated”* [older woman, El-Obeid].

*“There were many countries that stopped the vaccine, including some European Union countries, because it causes blood clots. But if it does not cause problems, [COVID-19] vaccination can be useful”* [older man, Damazin].

*“During this period, we got used to Corona. We are taking our precautions. At the moment, we are refusing to go get vaccinated”* [older woman, Tuti Island].

**Table 2:** Observed compliance with individual protective measures

	Adherence to COVID-19 prevention behaviors
Health Facilities	- Five sites included an observation session of a health centre in the neighbourhood. - Social distancing and individual protective measures such as mask-wearing and refraining from physical greetings were rarely implemented. - Persons wearing masks tend to remove them once they leave the health facility. - Most health professionals did not wear face masks or protective equipment or observe physical distancing.
Pharmacies	- Three sites included an observation session in a local pharmacy. - Physical distancing between customers was rarely observed. - Wearing of face coverings and avoidance of physical contact were rare among costumers - Mask-wearing was fairly common among pharmacists.
Places of worship	- All six sites included observation in a local mosque. - Few worshipers wore face masks, and few brought their prayer mats. - In all mosques, worshipers did not observe physical distancing during prayers and stood in lines shoulder to shoulder. - The majority of worshipers did not abstain from physical greetings.
Public Transportation	- All six sites included observation during a public bus trip. - Very few passengers wore face masks, whereas none of the drivers wore face masks. - No physical greetings were observed. - Physical distancing was not observed in all sites, with some passengers sharing seats. - In two of the sites, the bus station was overcrowded
Public markets	- All six sites included an observation session of a local public market. - Physical distancing between customers was rarely observed. - Wearing face coverings was rare (most wearers were women who covered their face using their traditional dress ‘toub’, and a few wore medical face masks). - Avoidance of physical contact was rare among customers and vendors. - Markets were crowded.
Bakeries	- All six sites included an observation session of a local bakery. - There were long queues of customers for hours. - Physical distancing was not observed in all sites, and it was rare to find customers wearing a mask or using hand sanitizer. - No avoidance of physical greetings.

### Restrictions on public gatherings and institutional implementation of prevention measures in communal spaces

The perceived importance of communal distancing measures among respondents who believe in COVID-19 was higher than reported and observed preventive measures in public areas. Implementing measures in public areas and bans on public gatherings were higher during the first wave and gradually stopped once severe movement restrictions were lifted. Mandates for restrictions on gatherings and implementation of prevention measures occurred mainly during the lockdown period. Outside this period, and without enforceable mandates, there were only rare examples in three communities (Tuti Island, Ombadda and Damazin) of private pharmacies or banks implementing social distancing measures. Based on observation of communal spaces in study sites, there were hardly any educational materials about COVID-19, hand washing or sanitisation stations, or markings on seats or the floor to facilitate social distancing in observed sites. Please see Table 3 for a detailed description of the implementation of communal prevention measures for COVID-19 in observed locations.

**Table 3:** Observed implementation of COVID-19 communal preventive measures

	Implementation of COVID-19 related health and safety measures
Health Facilities	- There were no stations for measuring the temperature of those entering the facilities or hand sanitizers in all facilities observed. - There were no markings on the floor or seating areas to indicate physical distancing. - Waiting areas in all but one health facility (Damazin) were poorly ventilated. - There were no educational flyers and posters about COVOD-19, social distancing, or the use of face masks in all facilities but one. - None of the health facilities had control over the number of visitors, and the waiting areas were crowded in all facilities observed
Pharmacies	- At two sites (Dereij and Gezira), there were educational posters about COVID-19 and written instructions mandating mask-wearing and physical distancing for customers. - Safe distancing was observed between pharmacists and customers. However, two sites did not have a physical barrier between customers and service providers. - Only one of the sites had a station for hand sanitizer.
Places of worship “Mosques”	- All mosques observed except one were closed, and they were opened 10 to 15 min before prayers and closed immediately after. - All mosques were kept clean and well ventilated either by keeping the windows open during prayers or using fans. - In all mosques except one (Tuti Island), there were no educational flyers and posters about COVID-19, no written instructions about physical distancing during prayers, bringing own holy book, using face masks or hand washing. - Imams included a speech on COVID-19 precautions in two of the observed mosques. - No stations for hand sanitizers, and handwashing stations did not have soap.
Public Transportation	- At all sites, buses were well-ventilated, and windows were open. - None of the buses had markings on seats to indicate physical distancing, and there were no educational flyers or posters about COVID-19, no written instructions about physical distancing or wearing masks in public transportation
Public markets	- All of the sites observed had no or few billboards about COVID-19 prevention. - There were no posters for written instructions mandating mask-wearing and physical distancing. - None of the markets observed except one (Tuti Island) had a dirty environment with safe distancing rarely observed. - There were no markings to indicate physical distancing, and handwashing and sanitizer stations were rare. - There were no physical barriers between customers and vendors.
Bakeries	- There were no educational posters about COVID-19 or written instruction mandating mask-wearing and social distancing. - There were no markings to indicate physical distancing, and handwashing and sanitizer stations were absent.

In general, most participants saw these measures in public spaces as supportive of or incentivising compliance with individual behaviors. However, most also felt that the onus was on the government and institutions providing services to mandate these measures and not depend on individuals’ decisions to comply.

*“If we say that this was left to people themselves to implement health precautions and they failed, the authorities need to have a role. For instance, the government needs to have a prompt response in restricting gatherings or obliging people to comply with protective measures in markets, mosques, or bakeries. The government needs to have an obvious role in this”* [older man, El-Obeid].

Generally, respondents had a low tolerance for the closure of mosques and bans on social events such as weddings and funerals. However, for a minority, such bans were considered a more tolerable alternative to lockdowns should COVID-19 cases and deaths increase.

### Barriers and facilitators of compliance with COVID-19 protective measures

There were barriers and facilitating factors to adherence to COVID-19 protective measures outside the household (See Table 4).

**Table 4:** Barriers and facilitators of adherence to COVID-19 protective measures in six Sudanese communities

	Facilitators of adherence to COVID-19 protective measures	Barriers to adherence to COVID-19 protective measures
i. Socioeconomic context	Youth seemingly more resilient/rebellious in the face of social pressures	Negative impact on business revenues and household income
	Supportive community measures and normalisation of new/adapted behaviors	Poverty and the high cost of essential supplies
		Shortages in necessities such as fuel and bread (long queues)
		The stigma associated with COVID-19 Bullying and harassment of compliers
		Denialism, rumors and misinformation about COVID-19 and the vaccine
ii. Cultural context	Cultural tendency to protect the vulnerable and the elderly	Preventive measures are seen as not compatible with Sudanese customs and habits
		Cultural tendency to bypass or manipulate government restrictions
iv. Religious beliefs	Recommendations to comply from well-regarded religious experts/institutions	Conflicts with scripture or religious practices (e.g. physical distancing between worshippers during prayer in mosques is seen as unacceptable)
v. Visibility of the pandemic and pandemic response	Enforcement of restrictions/protective measures in public areas and transportation means and penalties for non-compliers	
	Regular bulletins and information about COVD-19 in media	The waning intensity of awareness-raising activities
	Consistent and sustained risk communication activities (observed local languages and dialects and context-specific)	Mistrust in government information
	Visibility of pandemic locally (confirmed cases and deaths)	
vi. Feasibility	Provision of economic and in-kind support	Discomfort associated with mask-wearing
		Fatigue due to the long duration of the pandemic
vii. Perceived importance of behaviours and measures	Belief in the effectiveness of prevention measures	Inconvenience caused by compliance with protective measures in communal areas, e.g., long waiting times
	COVID-19 preventive behaviors and measures are also protective against other diseases	
viii. Perceived health system accessibility/capacity	Concerns because of difficulty in accessing health services	
	Recognizing the need to protect the weak health system from being overwhelmed with COVID-19 cases	

#### Socioeconomic and religious context

Due to a lack of compatibility with Sudanese customs and habits, such as community participation and collective interdependence, politeness, humility, generosity and hospitality, compliance was challenging with protective measures such as social distancing and acts of social withdrawal (stopping physical greetings, and not attending social events). However, the deeply-rooted Sudanese culture of protecting the vulnerable and duty of care towards the elderly motivated household members to comply with protective measures when outside the household. Participants also discussed that barriers include the absence of supportive measures in public areas, e.g. larger/ventilated public transportation, handwashing/sanitizers stations, and masks-wearing mandates. Socially, a discouraging attitude towards compliance with protective measures and the widespread stigma of COVID-19 prevailed, which created social pressure on compliers to abandon preventative measures.

*“My friend was harassed while using public transportation because she was wearing a mask; people literally walked behind her and verbally harassed her”* [younger woman, Gezira].

*“People would say you are arrogant, but this is not arrogance. I said to them that I am following the ministry of health guidelines. Also, one time I walked by and I said hi from a distance, they told me to come and greet them (physically), and so I told them that this illness has become common, so I won’t shake hands with them; they said to me “you are arrogant, and we no longer want to talk with you””* [older man, Ombadda].

Due to the current economic situation and hyperinflation, the high cost of supplies and competing priorities for expenditures hinder compliance with mask-wearing, hand sanitizers and frequent hand washing. Moreover, the economic costs of reducing working hours or opening hours for businesses were reported as an intolerable compromise. In addition, the limited availability of necessities, such as long queues in petrol stations and bakeries, meant people spent long hours outside the household and in crowded areas, making compliance with social distancing measures in public areas difficult.

The majority of the Sudanese population identify as Muslim, and people express their religious beliefs on a daily basis through regular prayers, including group prayers at mosques. According to participants in most study sites, social distancing during prayers in mosques and the closure of mosques during the pandemic were unacceptable compromises. However, due to trust in well-regarded religious scholars and institutions, social distancing during prayers can be implemented if supported by those entities.

*“Social distancing during prayer can be done, but it is honestly challenging; during prayers, it is difficult*” [older man, El-Obeid].

*“This (physical distancing during group prayers) needs consultations with religious experts. If a decision came from above, people would implement social distancing during prayer, no question”* [younger man, Damazin].

Another obstacle to compliance with protective measures was persistent COVID-19 denialism and rumors. According to participants, many rumors and misinformation were circulating about COVID-19. For instance, some participants mentioned that Africans are less prone to severe COVID-19, and others said the third wave was milder than the previous two waves. Another participant mentioned that the hot climate is protective as it weakens the virus.

*“Most housewives are scared of using hand sanitizer. Because it contains alcohol and if she used the gas cooker…there are number of fire casualties that happened because of this”* [younger woman, Damazin].

Other participants commented on a cultural tendency to bypass or manipulate restrictions.

*“I mean, you see a person sitting on a chair in a (telecommunications’ company) office, and when he gets up, you find written on the chair that is not allowed to sit on it”* [younger man, Ombadda].

#### Visibility of the pandemic and pandemic response

Increased visibility of the pandemic response locally and nationally was associated with increased risk perception levels and higher adherence to individual protective behaviors. Indicators of pandemic response mentioned by respondents included enforced restrictions or protective measures, presence of risk communication activities, compliance of local health workers with protective measures (mainly mask-wearing), and closure and opening of local isolation wards in their communities. Enforcement of restrictions and protective measures in communal areas and transportation means was mentioned as a facilitator for compliance. Some participants recommended the enforcement of penalties for non-compliers in public areas since, for many, a penalty was a primary driver of adherence, especially for physical distancing and mask-wearing.

*“I think that enforcement of restrictions support compliance with positive behaviors; also, penalties, even if small, for those who don’t comply can make people get used to these measures”* [older man, Damazin].

The participants confirmed that the reduced risk perception and diminished compliance with protective measures during the third wave were due to the lack of regular news coverage about COVID-19 and the waning of awareness-raising activities about the disease– both creating an impression that COVID-19 was no longer a threat. Furthermore, increased visibility of the pandemic locally was also associated with higher levels of perceived risk, increases in risk perception levels, and acceptance of bans on public gatherings (and vice versa). Participants’ indicators of an ongoing pandemic included personal experiences and losses due to COVID-19 and the overall number and trend of cases and deaths in their local communities.

#### Operational feasibility

The economic costs of masks, sanitisers, and reduced working hours when self-isolating were considered intolerable compromises for many people. Many mentioned the need for financial support to comply with these protective measures. Due to the long pandemic duration, many expressed their concerns about compliance fatigue, discomfort associated with mask-wearing and the harms of long-term use of hand sanitizers.

*“As my friend said, I do not wear a mask unless necessary. If I am not obliged to wear it, I would never wear it”* [older man, Gezira].

*“I need to know the side effects of these chemicals in the long term as I use it every 10 minutes. If we want to continue using sanitisers, they need to be environmentally friendly or have no chemicals because alcohol is strong on hands”* [younger woman, Tuti Island].

#### Perceived importance of behaviours and measures

Many participants reported that they complied with preventive measures to protect themselves, others and loved ones who are elderly or have chronic illnesses, reflecting their belief in the effectiveness of prevention measures. Furthermore, some participants mentioned that COVID-19 preventive measures are also protective against other diseases and that it is, therefore, efficient to comply. Other participants commented that compliance is vital to protect an already-weak health system from collapsing under the pressure of an overwhelming load of COVID-19 cases and having to increase health expenditures to cope.

*“Compliance with protective measures is essential, I swear, for all diseases not only corona, but one also needs to protect oneself and prevention is better than cure”* [younger woman, Ombadda].

*“It will also be helpful for the health system, I mean, corona make us pay more [health expenditure]”* [younger woman, El-Obeid].

Inconvenience caused by social distancing measures, such as long queues or waiting times, was reported as discouraging compliance to many people. Some participants mentioned they would refrain from using facilities that strictly enforce these measures if they could.

### Recommendations

#### Risk communication

To improve compliance with protective measures, participants recommended that the Ministry of Health have ongoing awareness-raising campaigns using multiple and parallel channels, such as championing local role models, religious sermons, giant billboards in public areas, and two-way dialogue methods like home visits. They also recommended having interventions that target the elderly tailored to their needs and lifestyle with practical tips for implementation. Participants emphasised the need to engage and train youth for awareness-raising activities in local communities and use local languages and dialects and context-specific messages.

#### Expectations of the government’s response

Many recommended that the government or the Ministry of Health provide regular and transparent updates about the COVID-19 situation nationally and locally to maintain a high level of public risk perception needed to increase compliance. Many participants mentioned specific expectations of the government’s role, such as enforcing physical distancing measures in public transportation and communal areas and ending shortages in necessities to eliminate lengthy and crowded queues at bakeries and other service facilities. Given the deteriorating economic situation in most people’s lives, participants also recommended governmental financial support for hard-hit families to support compliance. Finally, participants recommended accelerating COVID-19 vaccination campaigns to expedite the return to a pre-COVID state and reduce pressure on health services.

#### Recommendations for communities

Given the high perceived importance of protective behaviors and measures, study participants recommended that communities gear local efforts towards supporting masks wearing and using hand sanitizers/disinfectants for those most at risk of severe or fatal COVID-19. In addition, they emphasised the need to capitalise on emerging social habits such as shorter and smaller social events and no-contact greeting trends among the youth.

## Discussion

COVID-19 transmission is highly sensitive to the levels of social interaction and adherence to preventive measures. In the presence of low COVID-19 vaccine coverage in resource-poor settings [27], non-pharmaceutical interventions continue to be an essential pillar of the pandemic response. Our study explored changes in social contact patterns during an evolving COVID-19 pandemic in six communities in Sudan and identified barriers and enablers to adherence to selected individual and communal preventive measures. Understanding the evolution of the population’s interaction with the pandemic and pandemic response is essential to inform interventions by pandemic responders to encourage uptake of non-pharmaceutical interventions in the study communities.

We identified consistent trends in risk perception, social contacts and adherence to protective measures in our study population. As the pandemic progressed, restrictions were relaxed, the visibility of the pandemic response waned, social interactions increased, and risk perception and individual and institutional adherence to protective measures diminished. However, reported adherence to protective measures was consistently low throughout the pandemic, and adherence seemed to be primarily driven by the brief period of enforced movement restrictions. For example, reported mask use in Sudan, according to the University of Washington’s Institute for Health Metrics and Evaluation, was at its highest in December 2020, at 34% and dropped to 21% in April 2021 [28]. However, in contrast to our findings, a worldwide assessment of changes in adherence to COVID-19 protective behaviors reported low levels of initial adherence in similar low-income countries, followed by a later uptick in adherence [29]. Petherick et al. posit that adjustments in risk assessment by individuals may explain the behavioral changes they observed in the study. The assessment used changes in national COVID-19 case and death rates and citizens’ internet searches related to COVID-19 as proxies for variations in individuals’ risk perceptions. The assessment revealed a smaller rebound in adherence when controlling for these variations. In our study, we were able to contrast direct risk perception reports with adherence levels within each site and at different pandemic stages. Therefore, the absence of a later uptick in adherence to precautionary measures may be explained by individual risk assessments remaining unchanged after a decrease from initial higher levels of worry. Pandemic responders in Sudan need to increase the visibility of the local epidemic through strengthened surveillance and regular updates on the number of cases and deaths.

An individual’s appraisal of their capacity to adhere and sustain to adherence precautionary health behaviors influences their intent to adopt them [22]. We identified a lack of a supportive environment for protective behaviors, particularly once severe movement restrictions were lifted. Specifically, we identified social pressures that disincentivise adherence and extend to compliance-related bullying and harassment. Previous research has described COVID-19-related bullying directed towards COVID-19 patients [30] or healthcare workers [31]. However, evidence is scarce on the impact of bullying and harassment related to compliance with COVID-19 preventive measures in low-income settings. We also found that the limited affordability and availability of necessities meant that masks and sanitisers were perceived as inaccessible by many in the study population. This balancing act between the costs of, for example, masks and other necessities is experienced by populations in other African countries [32]. Despite these challenges, we identified a few instances of adaptations in social habits that are conducive to COVID-19 prevention. Pandemic responders in Sudan should acknowledge the social and economic pressures of preventive measures and use a solution-based approach in their risk communications activities. Responders should also actively promote and normalise new protective behaviors emerging in their communities.

Social and economic pressures in our study population were aggravated by persistent disbelief, rumors and misinformation about COVID-19. Research has shown that the public can easily distrust scientific evidence and believe rumors and conspiracy theories [33, 34]. Those who believe rumors and conspiracy theories are less likely to adopt health-protective behaviors such as wearing masks or social distancing [33, 35]. Pandemic responders in Sudan should actively track rumors and misinformation related to COVID-19 and COVID-19 vaccines in their communities and use tailored and context-specific interventions to address them.

Research has also shown that rumours and misinformation in other African countries tend to proliferate where there is low trust in government and mainstream media outlets [34]. There was widespread mistrust in government information about COVID-19 among our study population. An international survey examining the association of government trust regarding COVID-19 control with recommended health behaviours in 23 countries revealed that trust in governments is positively associated with the adoption of recommended prevention measures [36]. Participants in our study also implied that the government-led response was sub-optimal and largely invisible after the first epidemic wave, which further reduced their trust in the government. The same survey indicated that trust in governments during the COVID-19 pandemic, and hence compliance of the public with recommended health measures, was associated with perceived impartiality, transparency in conveying official statistics to the public, clear and organised information about COVID-19 and a perceived systematised response [34, 36]. Nonetheless, our study population had expectations of government action, including incentives, such as free distribution of masks and sanitizers and the enforcement of penalties for non-compliant individuals, public services and private institutions. The government should enforce incentive and penalty systems for individuals and institutions to both support those who intend to adopt preventive behaviors and increase the pandemic response’s visibility.

### Study strengths and limitations

Our study has strengths and limitations. Firstly, the timing of the data collection, not being during an active wave, would have influenced the current level of risk perception and implementation of prevention measures among targeted communities. Secondly, the competing priorities in the Sudanese context and possible pandemic fatigue might have affected the interest of the community members in the study. Data collectors were part of the communities, which helped reach the targeted participants. Having community members collecting the data was excellent in reaching communities and familiarity with the context. However, at the same time, that might have influenced the way they perceived information shared by the community members as being normal or taken for granted. Frequent discussions with team members from other sites and the research group were helpful. Finally, we acknowledge that the eligible age range for the older adult groups (25–59 years) is wide and cultural age-related dynamics may have affected the FGD dynamics between participants in these groups. The study has strengths that include a detailed description of the study context and methods, which can facilitate transferability to other similar contexts. Secondly, the triangulation of data collection methods observation and FGDs and triangulation of sources of information that included women and men and the different age groups provides a comprehensive understanding of the phenomenon.

## Conclusion

We identified generally low adherence to precautionary behaviours throughout the pandemic, driven primarily by low self-efficacy among the study population. However, we found variations in risk perception levels within and between study sites and at different pandemic stages. We argue that the unfavourable social and economic environment, coupled with the low visibility of the pandemic and pandemic response, have likely modulated the influence of risk perception on adopting precautionary behaviours by individuals, even in instances where risk perception was moderate or high. However, we also identified new social habits that can contribute to reducing COVID-19 transmission.

We recommend that COVID-19 responders increase the visibility of the local and national COVID-19 pandemic through consistent, regular and high-profile public updates on the number of cases and deaths. In addition, we recommend that governments and non-governmental actors increase the visibility of the pandemic response through continuous and appropriate health education interventions and highly-publicised coverage of treatment and vaccination programs. The government should also incentivise infection control measures in public areas and

health facilities. Rumors and misinformation related to COVID-19 and COVID-19 vaccines must be actively tracked and addressed through tailored interventions. Finally, we recommend interventions to actively normalise emerging healthy behaviours, such as shorter social gatherings and no-contact physical greetings.

### Electronic supplementary material

Below is the link to the electronic supplementary material.

Supplementary Material 1

Supplementary Material 2

Supplementary Material 3

## Data Availability

The datasets generated and analyzed during the current study are available from the corresponding author upon reasonable request.

## Abbreviations

COREQ: Consolidated Criteria for Reporting Qualitative Research
COVID-19: Coronavirus disease
IDP: Internally displaced persons
FGDs: Focus group discussions
Y-PEER Sudan: Sudan’s Youth Peer Education Network
