# Supplementary material for: Changes in social mixing and attitudes and practices to precautionary measures in a maturing COVID-19 pandemic in six communities in Sudan: a qualitative study
Source: BMC Public Health. 2024 Mar 26;24:895. doi: 10.1186/s12889-024-18274-7 (PMC10964503; doi:10.1186/s12889-024-18274-7)
Supplement: Supplementary file 1 — Supplementary Material 1 [file 12889_2024_18274_MOESM1_ESM.docx]

**Focus Group Discussion Guide**

**FGD no____ _____ Location________________**

**FGD participant group (select):**

**young girls and women / young boys and men / adult women / adult men**

**Date:______________________**

**Starting Time:_________________ Ending Time:________________**

RISK PERCEPTION (risk perception, change over time)

1. Are you worried about you getting COVID-19 infection?

Probing question (Is it the same level of anxiousness you feel now and during lockdown? If it changed, why is that? If it didn’t change, why is that?)

1. Are their certain people in your life that you are particularly worried about during the COVID-19 epidemic, or that you feel you need to protect? If yes, who are they and why are you concerned about them?

Probing question (Is it the same level of anxiousness you feel now and during lockdown? If it changed, why is that? If it didn’t change, why is that?)

SOCIAL CONTACT PATTERNS (change in patterns of social contacts outside home, comparison between three periods (before covid-19, movement restriction, now)

*We will now discuss social contacts that could transmit COVID-19 or other respiratory infections. These are close contacts which can be physical (such as a handshake, embracing, sharing a meal together out of the same bowl, playing football or other contact sports, sitting next to someone while touching shoulder to shoulder), or not physical, where you do not touch the person, but exchange at least a few words, face-to-face within 2 metres of them – for example, someone you bought something from in the market, or rode with on a bus or raksha, or worked with in the same area.*

Bearing this description in mind:

1. If we compare the period before the first COVID-19 case was reported in Sudan and the period of movement restriction (i.e. the first wave), how did your close contacts changed outside the household (within your neighbourhood, outside your HH, etc)? In which contexts did these contacts happen? Which gender did you interact mostly with? Which age group did you interact mostly with?
2. If we compare the period of lockdown and now, how did your close contacts changed outside the household (within your neighbourhood, outside your HH, etc)? In which contexts (work, social occasions, traveling etc.) did these contacts happen? Which gender did you interact mostly with? Which age group did you interact mostly with?
3. Generally, do you think that there has been a change in your social contacts since COVID-19 first appeared in Sudan compared to pre-COVID-19 state? If yes, in what way has it changed and why?

INDIVIDUAL PROTECTIVE BEHAVIOURS OUTSIDE THE HOME (significance, sustainability, compliance with behaviour, challenges to positive behaviour, facilitators of positive behaviour)

*We will now discuss recommended protective behaviours outside the home to prevent COVID-19 infection. These are: avoiding handshaking and physical greetings, face coverings, vaccination, safe space between self and others, avoiding close contact with people with COVID-19-like symptoms.*

1. Do you think it is important for people to maintain protective behaviours outside the home for example, in mosques, markets, bakeries, transportation, work etc.) to protect themselves from COVID-19? Why or why not?
2. Do you think it is possible to maintain these protective behaviours outside the home for several months? Why or why not? What makes it more difficult to do so? What makes it easier to do so?

We will now describe realistic scenarios for your related to these behaviours and we would like to hear your opinion on them:

1. Physical greetings:

Hajj Abdullah went to the mosque to pray. He met his neighbor Hajj Hassan, at the door of the mosque, and extended his hand in greeting. Hajj Hassan declined shaking hands, apologizing and reminding Hajj Abdullah of COVID-19 and the precautions that must be followed during this period. Hajj Abdullah was angry at his neighbour’s behaviour and did not accept this excuse, although he did not show it. What do you think of Hajj Hassan’s behavior and Hajj Abdullah’s reaction?

1. Face masks/covering:

While entering the hall where the neighborhood committee meeting is being held, Hoda put on her face mask and indicated to her colleague Safia to also wear a mask, while referring to the precautions that must be taken against COVID-19. Safia replied that she prefers not to wear the mask it makes her feel suffocated and because there are only a small number of attendees in the hall. What do you think about Hoda’s suggestion and Safia’s reaction?

1. Physical distancing:

Abdelrahman and his neighbor Ali went to buy bread from the nearby bakery in the neighborhood. The found a large crowd with no organisation of queues. Abdelrahman suggested that they should instead go to another bakery further away which had good organisation of queues with physical distancing, in line with precautions against COVID-19. Ali objected strongly to the suggestion, saying the other bakery was too far away, and insisting that they buy from the nearby bakery even if the customers were not abiding by physical distancing. What do you think of Abdulrahman’s suggestion and Ali’s response?

1. Vaccination:

As soon as Ibtisam returned home, she showed her grandmother the newspaper article that said they will soon start vaccination people in her age group against COVID-19. She indicated to her grandmother Hajja Sakina that she was very excited about this, and that tomorrow she will go to the local hospital to ask when she can arrange for her grandmother’s vaccination. Hajja Sakina told Ibtisam that she was not sure she wanted to get the vaccine since she doesn’t know what is in it and if it is safe. She also mentioned that COVID-19 seems to have now gone away so it was not necessary to get the vaccine. What do you think of Ibtisam’s suggestion and Hajja Sakina’s reaction?

1. Avoiding close contact with people with COVID-19-like symptoms:

While on a call with her friend Manal, Zainab mentioned that their neighbor, Fatima, is sick and has symptoms of COVID-19, and that they should go visit her and check on her. Manal replied saying that they should instead call Fatima on the phone rather than visit her, in keeping with precautions against COVID-19. Zainab then suggested that, to avoid blame from Fatima later on, they should go and visit her and just shorten the visit to a few minutes? Do you think they should go or not?

COMMUNAL DISTANCING MEASURES (what is happening, significance and sustainability)

*We will now discuss some of the recommended social distancing measures to prevent COVID-19 infection in communal facilities, such as mosques, markets, bakeries, transportation and other work places. For example, these include: signs and markers for physical distancing, handwashing/sanitising points, queues, work shifts. Other measures include restrictions on public gatherings such as weddings, funerals, demonstrations and sport events.*

1. Do communal areas in your community have social distancing measures? If yes, what kind of measures have you observed?
2. Do you think it is important for communal areas to put measures for physical distancing in place? Why or why not?
3. Do you think it is possible for communal areas in your community to maintain such measures for several months?
4. Are public gatherings currently occurring in your community? If yes, what kind of gatherings?
5. Do you think it is important to restrict public gatherings and why?
6. Do you think it is possible to restrict public gatherings for several months and why?

RECOMMENDATIONS

1. What are your recommendations for encouraging and supporting people to successfully adopt and maintain protective behaviours outside the home for COVID-19 prevention?

CONCLUSION

1. Are there any other thoughts you would like to share with us or with the group?

Thank you all for your participation, we will get in touch to arrange for another discussion in few weeks.
