# Supplementary material for: Changes in social mixing and attitudes and practices to precautionary measures in a maturing COVID-19 pandemic in six communities in Sudan: a qualitative study
Source: BMC Public Health. 2024 Mar 26;24:895. doi: 10.1186/s12889-024-18274-7 (PMC10964503; doi:10.1186/s12889-024-18274-7)
Supplement: Supplementary file 2 — Supplementary Material 2 [file 12889_2024_18274_MOESM2_ESM.docx]

**Structured Observation Guides**

**Health facilities**

**Name of observer: ________________ Location:____________________**

**Date:______________________ Starting time:_________________ Ending Time:________________**

**Implementation of COVID-19 related health and safety measures**

| **Measure** | **Yes** | **No** | **Partially** | **Description/Comments** |
| --- | --- | --- | --- | --- |
| Temperature checks at the entrance of the facility |  |  |  |  |
| Health facility staff are wearing face coverings or medical masks |  |  |  |  |
| Hand sanitizer or soap and water at handwashing stations are freely available and accessible to all visitors and staff |  |  |  |  |
| General cleanliness of the facility |  |  |  |  |
| A person is available to organise entrance to the facility, control the number of people in the facility or waiting rooms, and/or organise queues |  |  |  |  |
| Presence of visual signs or markers for safe physical distancing between visitors in waiting areas and at the entrance of the facility |  |  |  |  |
| Presence of visual materials (e.g. posters) that raise awareness of COVID-19 |  |  |  |  |

**Further notes:**

**……………………………………………………………………………………………………………………………………………………………………………………………………………………………………………………………………………………………………………………………………………………………………**

**Adherence to COVID-19 prevention behaviours**

| **Behaviour** | **Common** | **Rare** | **Absent** | **Description/Comments** |
| --- | --- | --- | --- | --- |
| Wearing of face coverings or medical masks (staff and visitors) |  |  |  |  |
| Abstaining from physical greetings (staff and visitors) |  |  |  |  |
| Observing safe space from others at entrance and/or waiting areas (staff and visitors) |  |  |  |  |
| Any other incidental behavioural observations relevant to COVID-19  (e.g. cough etiquette, hand sanitising, handwashing, etc…)  (staff and visitors) |  |  |  |  |

**Further notes:**

**………………………………………………………………………………………………………………………………………………………………………………………………………………………………………………………………………………………………………………………………………………………………………………**

**Description of people present in the observed location**

| **Description** | **Many** | **Some** | **Few** | **Absent** | **Description/Comments** |
| --- | --- | --- | --- | --- | --- |
| **Age and gender** | | | | | |
| Babies, young children, children up to (0 - 10 years) |  |  |  |  |  |
| Males |  |  |  |  |  |
| Females |  |  |  |  |  |
| Adolescents and youth (10- 24 years) |  |  |  |  |  |
| Males |  |  |  |  |  |
| Females |  |  |  |  |  |
| Adults (25 – 59 years) |  |  |  |  |  |
| Males |  |  |  |  |  |
| Females |  |  |  |  |  |
| Elderly (60 years +) |  |  |  |  |  |
| Males |  |  |  |  |  |
| Females |  |  |  |  |  |
| Purpose of visit (based on observations) |  |  |  |  |  |
| Staff |  |  |  |  |  |
| Patients |  |  |  |  |  |
| Co-patients |  |  |  |  |  |
| Others (e.g. street beggars, etc.) |  |  |  |  |  |

**Further notes:**

**…………………………………………………………………………………………………………………………………………………………………………………………………………………………………………………………………………………………………………………………………………………………………………….**

**Brief description of the environment in the observation location (e.g. weather, location relative to the geographical centre of the study site, nearby places of congregation such as a university, school or police station, presence of protests or civil unrest, etc.):**

**………………………………………………………………………………………………………………………………………………………………………………………………………………………………………………………………………………………………………………………………………………………………………….**

**Places of worship (mosque or church)**

**Name of observer: ________________ Location:____________________**

**Date:______________________ Starting time:_________________ Ending Time:________________ Prayer: ______________________**

1. **Implementation of COVID-19 related health and safety measures**

| **Measure** | **Yes** | **No** | **Partially** | **Description/Comments** |
| --- | --- | --- | --- | --- |
| Opening and closing the mosque or church 15 mins before and after prayers |  |  |  |  |
| Opening windows and good ventilation indoors, or prayer in outside areas |  |  |  |  |
| Presence of a guide or visible guidance for mask wearing or face coverings and/or maintaining physical distancing at entrance/exit. |  |  |  |  |
| In a mosque: Presence of visual guidance for visitors to bring and use their own prayer mats |  |  |  |  |
| Presence of visual guidance or verbal instructions for worshippers to maintain a 1.5m – 2m distance from each other during prayer |  |  |  |  |
| Hand sanitizer or soap and water at handwashing/ablution stations are freely available and accessible to all visitors |  |  |  |  |
| General cleanliness of the facility |  |  |  |  |
| Presence of visual guidance or verbal instructions for worshippers to leave a vacant row between every two rows of worshippers during prayers |  |  |  |  |
| Removal of all holy books from the mosque or church (Mushaf or Bible) and visual guidance or verbal instructions for worshippers to bring and user their own books |  |  |  |  |
| Remove/disconnect water fountains and ban eating and drinking in the mosque/church |  |  |  |  |
| Presence of visual materials (e.g. posters) that raise awareness of COVID-19 |  |  |  |  |
| After-prayer sermons by imam or priest to raise awareness about COVID-19 |  |  |  |  |

**Further notes:**

**……………………………………………………………………………………………………………………………………………………………………………………………………………………………………………………………………………………………………………………………………………………………………**

1. **Adherence to COVID-19 prevention behaviours**

| **Behaviour** | **Common** | **Rare** | **Absent** | **Description/Comments** |
| --- | --- | --- | --- | --- |
| Wearing of face coverings or medical masks |  |  |  |  |
| Abstaining from physical greetings |  |  |  |  |
| Observing safe space from others at entrance |  |  |  |  |
| Observing safe space from other during prayers |  |  |  |  |
| Use of personal prayer mats and/or holy books |  |  |  |  |
| Avoiding eating and drinking in the mosque/church |  |  |  |  |
| Any other incidental behavioural observations relevant to COVID-19  (e.g. cough etiquette, hand sanitising, handwashing, etc…) |  |  |  |  |

**Further notes:**

**………………………………………………………………………………………………………………………………………………………………………………………………………………………………………………………………………………………………………………………………………………………………………………**

1. **Description of people present in the observed location**

| **Description** | **Many** | **Some** | **Few** | **Absent** | **Description/Comments** |
| --- | --- | --- | --- | --- | --- |
| **Age and gender** | | | | | |
| Babies, young children, children up to (0 - 10 years) |  |  |  |  |  |
| Males |  |  |  |  |  |
| Females |  |  |  |  |  |
| Adolescents and youth (10- 24 years) |  |  |  |  |  |
| Males |  |  |  |  |  |
| Females |  |  |  |  |  |
| Adults (25 – 59 years) |  |  |  |  |  |
| Males |  |  |  |  |  |
| Females |  |  |  |  |  |
| Elderly (60 years +) |  |  |  |  |  |
| Males |  |  |  |  |  |
| Females |  |  |  |  |  |
| Purpose of visit (based on observations) |  |  |  |  |  |
| Staff |  |  |  |  |  |
| Worshippers |  |  |  |  |  |
| Others (e.g. street beggars, etc.) |  |  |  |  |  |

**Further notes:**

**………………………………………………………………………………………………………………………………………………………………………………………………………………………………………………………………………………………………………………………………………………………………………………**

1. **Brief description of the environment in the observation location (e.g. weather, location relative to the geographical centre of the study site, nearby places of congregation such as a university, school or police station, presence of protests or civil unrest, etc.):**

**………………………………………………………………………………………………………………………………………………………………………………………………………………………………………………………………………………………………………………………………………………………………………….**

**Public transportation**

**Name of observer: ________________ Location:____________________**

**Date:______________________ Starting time:_________________ Ending Time:________________**

1. **Implementation of COVID-19 related health and safety measures**

| **Measure** | **Yes** | **No** | **Partially** | **Description/Comments** |
| --- | --- | --- | --- | --- |
| Opening windows and good ventilation |  |  |  |  |
| Presence of a guide or visible guidance for mask wearing or face coverings |  |  |  |  |
| Presence of a guide or visible guidance for maintaining physical distancing while seated, for example, leaving vacant seats between passengers. |  |  |  |  |
| General cleanliness of the vehicle |  |  |  |  |
| Presence of visual materials (e.g. posters) that raise awareness of COVID-19 |  |  |  |  |

**Further notes:**

**……………………………………………………………………………………………………………………………………………………………………………………………………………………………………………………………………………………………………………………………………………………………………**

1. **Adherence to COVID-19 prevention behaviours**

| **Behaviour** | **Common** | **Rare** | **Absent** | **Description/Comments** |
| --- | --- | --- | --- | --- |
| Wearing of face coverings or medical masks |  |  |  |  |
| Abstaining from physical greetings |  |  |  |  |
| Observing safe space from others at entrance |  |  |  |  |
| Observing safe space from other while seated |  |  |  |  |
| Any other incidental behavioural observations relevant to COVID-19  (e.g. cough etiquette, hand sanitising, money-handling, etc…) |  |  |  |  |

**Further notes:**

**……………………………………………………………………………………………………………………………………………………………………………………………………………………………………………………………………………………………………………………………………………………………………**

1. **Description of people present in the observed location**

| **Description** | **Many** | **Some** | **Few** | **Absent** | **Description/Comments** |
| --- | --- | --- | --- | --- | --- |
| **Age and gender** | | | | | |
| Babies, young children, children up to (0 - 10 years) |  |  |  |  |  |
| Males |  |  |  |  |  |
| Females |  |  |  |  |  |
| Adolescents and youth (10- 24 years) |  |  |  |  |  |
| Males |  |  |  |  |  |
| Females |  |  |  |  |  |
| Adults (25 – 59 years) |  |  |  |  |  |
| Males |  |  |  |  |  |
| Females |  |  |  |  |  |
| Elderly (60 years +) |  |  |  |  |  |
| Males |  |  |  |  |  |
| Females |  |  |  |  |  |

**Further notes:**

**……………………………………………………………………………………………………………………………………………………………………………………………………………………………………………………………………………………………………………………………………………………………………**

1. **Brief description of the environment in the observation location (e.g. weather, location relative to the geographical centre of the study site, nearby places of congregation such as a university, school or police station, presence of protests or civil unrest, etc.):**

**………………………………………………………………………………………………………………………………………………………………………………………………………………………………………………………………………………………………………………………………………………………………………….**

**Pharmacies**

**Name of observer: ________________ Location:____________________**

**Date:______________________ Starting time:_________________ Ending Time:________________**

1. **Implementation of COVID-19 related health and safety measures**

| **Measure** | **Yes** | **No** | **Partially** | **Description/Comments** |
| --- | --- | --- | --- | --- |
| Opening windows and good ventilation |  |  |  |  |
| Presence of a guide or visible guidance for mask wearing or face coverings |  |  |  |  |
| Presence of a guide or visible guidance for maintaining physical distancing |  |  |  |  |
| Presence of physical barriers for infection prevention when serving customers |  |  |  |  |
| Hand sanitizer freely available and accessible to all visitors |  |  |  |  |
| General cleanliness of the pharmacy |  |  |  |  |
| Presence of visual materials (e.g. posters) that raise awareness of COVID-19 |  |  |  |  |

**Further notes:**

**……………………………………………………………………………………………………………………………………………………………………………………………………………………………………………………………………………………………………………………………………………………………………**

1. **Adherence to COVID-19 prevention behaviours**

| **Behaviour** | **Common** | **Rare** | **Absent** | **Description/Comments** |
| --- | --- | --- | --- | --- |
| Wearing of face coverings or medical masks |  |  |  |  |
| Abstaining from physical greetings |  |  |  |  |
| Observing safe space from others at the entrance or inside the pharmacy |  |  |  |  |
| Any other incidental behavioural observations relevant to COVID-19  (e.g. cough etiquette, hand sanitising, money-handling, etc…) |  |  |  |  |

**Further notes:**

**……………………………………………………………………………………………………………………………………………………………………………………………………………………………………………………………………………………………………………………………………………………………………**

1. **Description of people present in the observed location**

| **Description** | **Many** | **Some** | **Few** | **Absent** | **Description/Comments** |
| --- | --- | --- | --- | --- | --- |
| **Age and gender** | | | | | |
| Babies, young children, children up to (0 - 10 years) |  |  |  |  |  |
| Males |  |  |  |  |  |
| Females |  |  |  |  |  |
| Adolescents and youth (10- 24 years) |  |  |  |  |  |
| Males |  |  |  |  |  |
| Females |  |  |  |  |  |
| Adults (25 – 59 years) |  |  |  |  |  |
| Males |  |  |  |  |  |
| Females |  |  |  |  |  |
| Elderly (60 years +) |  |  |  |  |  |
| Males |  |  |  |  |  |
| Females |  |  |  |  |  |
| Purpose of visit (based on observations) |  |  |  |  |  |
| Staff |  |  |  |  |  |
| Customers |  |  |  |  |  |
| Others (e.g. street beggars, etc.) |  |  |  |  |  |

**Further notes:**

**……………………………………………………………………………………………………………………………………………………………………………………………………………………………………………………………………………………………………………………………………………………………………**

1. **Brief description of the environment in the observation location (e.g. weather, location relative to the geographical centre of the study site, nearby places of congregation such as a university, school or police station, presence of protests or civil unrest, etc.):**

**…………………………………………………………………………………………………………………………………………………………………………………………………………………………………………………………………………………………………………………………………………………………………**

**Markets**

**Name of observer: ________________ Location:____________________**

**Date:______________________ Starting time:_________________ Ending Time:________________**

1. **Implementation of COVID-19 related health and safety measures**

| **Measure** | **Yes** | **No** | **Partially** | **Description/Comments** |
| --- | --- | --- | --- | --- |
| Presence of handwashing stations with soap and water that are freely available and accessible to all customers and traders |  |  |  |  |
| Presence of visible guidance for mask wearing or face coverings |  |  |  |  |
| Presence of visible guidance for maintaining physical distancing |  |  |  |  |
| Presence of physical barriers for infection prevention when serving customers |  |  |  |  |
| General cleanliness of the market |  |  |  |  |
| Presence of visual materials (e.g. posters) that raise awareness of COVID-19 |  |  |  |  |

**Further notes:**

**……………………………………………………………………………………………………………………………………………………………………………………………………………………………………………………………………………………………………………………………………………………………………**

1. **Adherence to COVID-19 prevention behaviours**

| **Behaviour** | **Common** | **Rare** | **Absent** | **Description/Comments** |
| --- | --- | --- | --- | --- |
| Wearing of face coverings or medical masks |  |  |  |  |
| Abstaining from physical greetings |  |  |  |  |
| Observing safe space from others |  |  |  |  |
| Any other incidental behavioural observations relevant to COVID-19  (e.g. cough etiquette, hand sanitising, money-handling, etc…) |  |  |  |  |

**Further notes:**

**……………………………………………………………………………………………………………………………………………………………………………………………………………………………………………………………………………………………………………………………………………………………………**

1. **Description of people present in the observed location**

| **Description** | **Many** | **Some** | **Few** | **Absent** | **Description/Comments** |
| --- | --- | --- | --- | --- | --- |
| **Age and gender** | | | | | |
| Babies, young children, children up to (0 - 10 years) |  |  |  |  |  |
| Males |  |  |  |  |  |
| Females |  |  |  |  |  |
| Adolescents and youth (10- 24 years) |  |  |  |  |  |
| Males |  |  |  |  |  |
| Females |  |  |  |  |  |
| Adults (25 – 59 years) |  |  |  |  |  |
| Males |  |  |  |  |  |
| Females |  |  |  |  |  |
| Elderly (60 years +) |  |  |  |  |  |
| Males |  |  |  |  |  |
| Females |  |  |  |  |  |
| Purpose of visit (based on observations) |  |  |  |  |  |
| Shop traders |  |  |  |  |  |
| Street traders (e.g. tea sellers, book sellers, etc.) |  |  |  |  |  |
| Customers |  |  |  |  |  |
| Others (e.g. street beggars, etc.) |  |  |  |  |  |

**Further notes:**

**……………………………………………………………………………………………………………………………………………………………………………………………………………………………………………………………………………………………………………………………………………………………………**

1. **Brief description of the environment in the observation location (e.g. weather, location relative to the geographical centre of the study site, nearby places of congregation such as a university, school or police station, presence of protests or civil unrest, etc.):**

**………………………………………………………………………………………………………………………………………………………………………………………………………………………………………………………………………………………………………………………………………………………………………….**

**Bakeries**

**Name of observer: ________________ Location:____________________**

**Date:______________________ Starting time:_________________ Ending Time:________________**

1. **Implementation of COVID-19 related health and safety measures**

| **Measure** | **Yes** | **No** | **Partially** | **Description/Comments** |
| --- | --- | --- | --- | --- |
| Presence of visible guidance for mask wearing or face coverings |  |  |  |  |
| Presence of a guide to organise queues |  |  |  |  |
| Presence of visible guidance for maintaining physical distancing |  |  |  |  |
| Presence of physical barriers for infection prevention when serving customers |  |  |  |  |
| Hand sanitizer freely available and accessible to all visitors |  |  |  |  |
| General cleanliness of the bakery |  |  |  |  |
| Presence of visual materials (e.g. posters) that raise awareness of COVID-19 |  |  |  |  |

**Further notes:**

**……………………………………………………………………………………………………………………………………………………………………………………………………………………………………………………………………………………………………………………………………………………………………**

1. **Adherence to COVID-19 prevention behaviours**

| **Behaviour** | **Common** | **Rare** | **Absent** | **Description/Comments** |
| --- | --- | --- | --- | --- |
| Wearing of face coverings or medical masks |  |  |  |  |
| Abstaining from physical greetings |  |  |  |  |
| Observing safe space from others at the entrance or inside the bakery |  |  |  |  |
| Any other incidental behavioural observations relevant to COVID-19  (e.g. cough etiquette, hand sanitising, money-handling, etc…) |  |  |  |  |

**Further notes:**

**……………………………………………………………………………………………………………………………………………………………………………………………………………………………………………………………………………………………………………………………………………………………………**

1. **Description of people present in the observed location**

| **Description** | **Many** | **Some** | **Few** | **Absent** | **Description/Comments** |
| --- | --- | --- | --- | --- | --- |
| **Age and gender** | | | | | |
| Babies, young children, children up to (0 - 10 years) |  |  |  |  |  |
| Males |  |  |  |  |  |
| Females |  |  |  |  |  |
| Adolescents and youth (10- 24 years) |  |  |  |  |  |
| Males |  |  |  |  |  |
| Females |  |  |  |  |  |
| Adults (25 – 59 years) |  |  |  |  |  |
| Males |  |  |  |  |  |
| Females |  |  |  |  |  |
| Elderly (60 years +) |  |  |  |  |  |
| Males |  |  |  |  |  |
| Females |  |  |  |  |  |
| Purpose of visit (based on observations) |  |  |  |  |  |
| Staff |  |  |  |  |  |
| Customers |  |  |  |  |  |
| Others (e.g. street beggars, etc.) |  |  |  |  |  |

**Further notes:**

**……………………………………………………………………………………………………………………………………………………………………………………………………………………………………………………………………………………………………………………………………………………………………**

1. **Brief description of the environment in the observation location (e.g. weather, location relative to the geographical centre of the study site, nearby places of congregation such as a university, school or police station, presence of protests or civil unrest, etc.):**

**…………………………………………………………………………………………………………………………………………………………………………………………………………………………………………………………………………………………………………………………………………………………………………**
